# Integrated optical beam steering device using switchable nanoantennas and a reflective metalens

**DOI:** 10.1038/s41598-023-33939-w

**Published:** 2023-05-02

**Authors:** Vahid Ghaffari, Leila Yousefi

**Affiliations:** School of Electrical and Computer Engineering, College of Engineering, University of Tehran, Tehran, 1417614411 Iran

**Keywords:** Electrical and electronic engineering, Nanophotonics and plasmonics, Photonic devices

## Abstract

In this paper, an integrated optical device is proposed in which a reflective meta-lens and five switchable nano-antennas are combined to provide optical beam steering at the standard telecommunication wavelength of 1550 nm. For this purpose, a graphene-based switchable power divider is designed and integrated with nano-antennas to control the flow of the light entering the device. To achieve a higher angular accuracy in the radiated beams, a new algorithm is proposed and utilized to optimize the location of feeding nano-antennas in accordance with the reflective meta-lens. In order to achieve a minimum fluctuation in the light intensity when the beams are rotated in the space, an algorithm is developed to select optimum unit cells for the engineered meta-lens. The whole device is numerically analyzed using Electromagnetic full-wave simulations illustrating an optical beam steering with high accuracy (better than 1 degree) in the beam direction, and a low variation (less than 1 dB) in the radiated light intensity. The proposed integrated device can be used for many applications such as inter- and intra-chip optical interconnects, optical wireless communication systems, and advanced integrated LIDARs.

## Introduction

Optical nano-antennas are devices designed to control the light profile in micro and nanometer dimensions^1–4^. Their ability to control the light can be used in many different applications including optical wireless communication system^5–8^, plasmonic biosensors^9^, sub-wavelength imaging instruments^10–12^, and also light trapping in solar cells^13,14^. Dynamic control of the radiation pattern of nano-antennas, called the beam steering capability, can provide more flexibility in before mentioned applications specially when using them for authentication^15^, optical communication^6^, holography^16^, imaging^17^ and LIDARs ^18,19^.

To realize optical beam steering, different methods including phase array antennas^20–23^, leaky wave antennas^27–32^, and metasurfaces with tunable unit cells^33–38^ have been proposed so far. However, all of the previously developed techniques have their own limitations and disadvantages that make developing new techniques and methods to realize optical beam steering an ongoing research stream.

Phased array antennas, which are used widely in microwave regime to provide beam scanning, consist of a set of identical optical nano-antennas in which the beam is controlled by adjustable phase shifters connected to each antenna element. Narrow beam width, wide beam scanning and high resolution are the advantages of optical phased array antennas. However, some limitations and disadvantages such as slow adjustable phase shifters^20^, large dimensions^20–23^, and high level of annoying lobes^22,23^ restrict their applications. The integrated structures, equipped with a Luneburg lens^24^ or Rotman lens^25^, don’t require phase shifters and enable the beam steering over a wide scanning range. However, they suffer from high loss and fabrication complexity^24–26^.

In another approach, leaky wave structures are used to reduce the dimensions and eliminate the need for phase shifters. These structures can be categorized into single and multi-tone groups. In multi-tone leaky wave antennas, the rotation of the beam is achieved by changing the radiation wavelength which requires access to expensive and high-bandwidth lasers^27–30^. Single-tone structures, however, operate based on variation of the refractive index over a single wavelength. In this method, the refractive index is mainly changed thermally which makes it to be a low-speed technique^31,32^. In addition, small field of view (FOV) and high loss can be considered as other disadvantages of leaky wave antennas^27–32^.

Another way to steer radiated beams is using tunable metasurfaces^33–38^. Metasurfaces are two-dimensional version of metamaterials consisting of a set of nano-antennas each providing specific reflected amplitude and phase. Tunable materials such as vanadium dioxide (VO_2_)^33,34^, Indium tin oxide (ITO)^35,36^, and phase-change materials (PCMs)^37,38^ can be used in construction of metasurfaces, making their response dynamically controllable. Tunable metasurfaces when used for beam steering provide narrow radiation beam, wide FOV, and relatively high-speed steering. However, since each unit cell used in construction of metasurfaces should be tuned individually, it increases the complexity and cost of these structures^33–38^. To overcome this problem, lens-based structures have been introduced to work in microwave^57–59^ and optical^49–55^ regime.

In order to address the challenges mentioned above, in this paper, an integrated device is proposed in which an array of nano-antennas, a graphene-based switchable power divider, and a reflective metasurface are combined together to provide optical beam steering. All elements are integrated inside a silicon dioxide medium, making a compact device having dimensions of $10.2\times 16.3\times 6.5 \,  \upmu {\text{m}}^{3}$. The structure is designed in such a way that it can be fabricated with standard nano-technology techniques. The beam steering is provided by switching among feeding nano-antennas realized by designed and optimized graphene-based switchable power divider operating based on applied voltages that control the chemical potential of graphene sheets. Since the beam steering is realized electronically, the device has a higher speed in comparison with the designs and methods that control the radiation beam mechanically or thermally. Since the metasurface used in this design is not tunable, to avoid the complexity in fabrication and control, one of the most challenging parts was designing unit cells that simultaneously provide different suitable phases for different feeding nano-antennas. To address this challenge, a new method is proposed which uses holography technique to calculate required phases and then by defining a suitable phase error function, selects optimum locations for feeding nano-antennas that minimize the defined function. The whole structure is numerically analyzed and its performance is investigated using Electromagnetic full-wave simulations. The results of this simulation, show several advantages for the designed structure, when compared to previously reported works, including high accuracy for the designed radiation angles, low side-lobe levels and low variation in the radiated power intensity when performing beam steering.

The structure of the paper is as follows. First, the proposed structure is presented and its principle of operation is explained. In this part of the paper, the components constructing the device, nano-antennas, metasurface unit cells and graphene-based switch, are described individually and their performances are numerically investigated one by one. Furthermore, the algorithm used to calculate the phase required from metasurface unit cells, and to find the optimum locations of feeding nano-antennas to minimize the resultant phase error, is explained. Then, the whole proposed beam steering device is numerically analyzed and its results when used for beam steering are presented and discussed. In that part, also the ability of the proposed structure to be extended in order to achieve higher resolution and narrower beam is investigated. Finally in the last section, we conclude the paper.

## Proposed structure and its principle of operation

**Figure 1:**
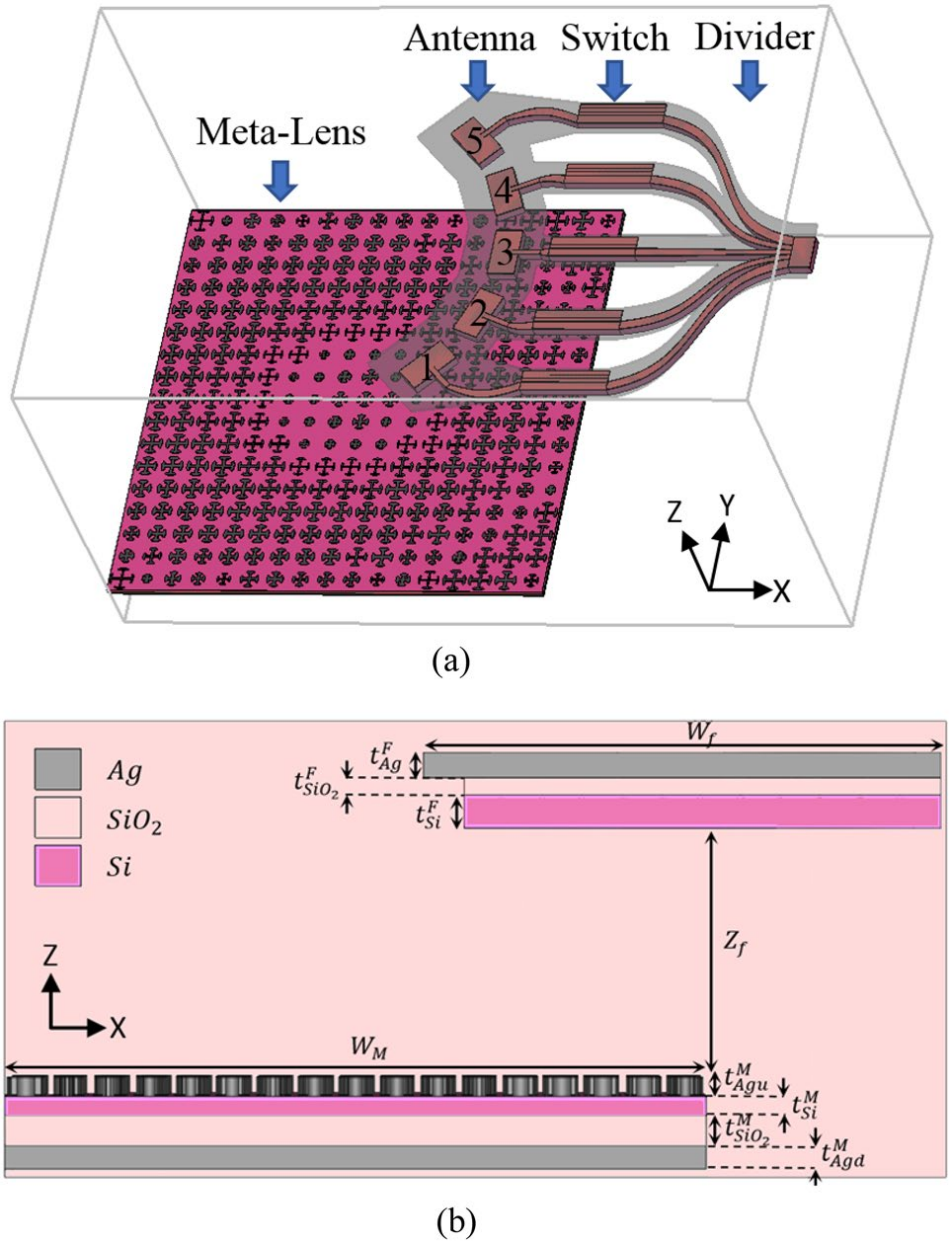
(**a**) Perspective view (**b**) Side view of the proposed beam steering device consisting of a reflective meta-lens, feeding nano-antennas, and graphene-based switchable power divider. The dimensions scales are not in proportion. ${W}_{m}=9.6  \,  \upmu {\text{m}}, \, {t}_{Agu}^{M}=20 \,  {\text{nm}}, \, {t}_{Agd}^{M}=50 \,  {\text{nm}}, \, {t}_{{SiO}_{2}}^{M}=100 \,  {\text{nm}}, \, {t}_{Si}^{M}=10 \,  {\text{nm}},{W}_{f}=10.1  \,  \upmu {\text{m}}, \, {Z}_{f}=6.05  \,  \upmu {\text{m}}, \, {t}_{Ag}^{F}=100 \,  {\text{nm}}, \, {{t}_{{SiO}_{2}}^{F}=20 \,  {\text{nm}}, t}_{Si}^{F}=150 \,  {\text{nm}}$.

The proposed integrated beam steering device is shown in Fig. 1. As shown in this figure, the designed structure consists of a metasurface-based lens integrated with five nano-antennas connected to a graphene-based optical switch. The whole structure has a dimension of $10.2\times 16.3\times 6.5 \,  \upmu {\text{m}}^{3}$ and is integrated inside silicon dioxide, acting as the background material. The device is designed in such a way that it can be fabricated using standard nanotechnology fabrication techniques. The meta-lens consists of $17\times 17$ plasmonic unit cells, constructed from silicon and SiO_2_ layers sandwiched between two layers of Silver.

The radiated beam that is coming out of the device is mainly provided by the reflective metasurface-based lens. This meta-lens shapes the incoming light illuminated on it by the nano-antennas, to provide the radiation in the desired direction. The meta-lens is designed in such a way that it provides radiation in different directions when illuminated by different feeds. Therefore, the beam steering is provided by selecting among the radiated nano-antennas. This selection is realized using a controllable graphene-based power divider (see Fig. 1), by applying appropriate voltages to the graphene layers used in its construction. At first, the incoming light enters the graphene-based switchable power divider, and according to the adjustable voltages applied to the switch, it is guided to one of the five output ports feeding the related nano-antenna. The selected nano-antenna fed by the switch will radiate the light normally to the space. The radiated light is shined on the meta-lens placed in front of antennas, and then reflected by the lens to a specific direction. In the following, we provide further details on each component of the proposed structure.

### Metasurface-based reflecting lens

To achieve a high accuracy beam steering, the meta-lens and locations of the feeding nano-antennas are designed using holography method^39–48^. In the classical holography method, incident wave produced by one source and the other wave obtained from scattering of an object interfere on the hologram, and their interference pattern is recorded on a photographic film^48^. Then, illumination of the film with the reference wave, will scatter a copy of the original object wave. The interference pattern on the hologram contains a term proportional to^48^:

1 $$H\left({x}^{^{\prime}},{y}^{^{\prime}}\right)={\bar{\psi }}_{o}\left({x}^{^{\prime}},{y}^{^{\prime}}\right) \cdot {\bar{\psi }}_{i}^{*}\left({x}^{^{\prime}},{y}^{^{\prime}}\right)$$

where ${\psi }_{i}$ is the incident wave, ${\psi }_{o}$ is the object wave and $H$ is the desired hologram pattern. When the recorded hologram is illuminated by the incident wave, the scattered wave from the hologram gives $H\left({x}^{^{\prime}},{y}^{^{\prime}}\right).{\psi }_{i}\left({x}^{^{\prime}},{y}^{^{\prime}}\right)={\psi }_{o}\left({x}^{^{\prime}},{y}^{^{\prime}}\right)|{\psi }_{i}^{2}\left({x}^{^{\prime}},{y}^{^{\prime}}\right)|$ which is a copy of the object wave. When using holography technique for designing meta-lenses, the ${\psi }_{i}$ represents the light incident on the meta-lens, the $H$ represents the response of the meta-lens which is provided by proper design of unit cells, and (${\psi }_{o}$) represents the desired reflected pattern.

In this design, the incident wave, ${\overline{\psi }}_{i}$, is determined by the feeding nano-antennas. Therefore, by assumption of TM polarization for the incident and scattered waves, the ${\overline{\psi }}_{i}$, can be written as:

2 $${\bar{\psi }}_{i}\left({x}^{^{\prime}},{y}^{^{\prime}}\right)={A}_{i}\left(\mathrm{cos}{\theta }_{i}\; cos{\varphi }_{i} \; \widehat{x}+\mathrm{cos}{\theta }_{i} \; sin{\varphi }_{i} \; \widehat{y}\right) {e}^{j{k}_{0}|\bar{R{^{\prime}}}-{\overline{R} }_{i}|}$$

where ${A}_{i}$, is the amplitude of the incident wave on the meta-lens, ${k}_{0}$ is the free space wave number, ${\overline{R} }_{i}$ is a vector connecting the center of the meta-lens to the nano-antenna location and can be written as ${\overline{R} }_{i}={R}_{i}\mathrm{sin}{\theta }_{i}cos{\varphi }_{i} \widehat{x}+ {R}_{i}\mathrm{sin}{\theta }_{i}sin{\varphi }_{i} \widehat{y}+ {R}_{i}\mathrm{cos}{\theta }_{i} \widehat{z}$, and ${\overline{R} }^{^{\prime}}={x}^{^{\prime}}\widehat{x}+{y}^{^{\prime}}\widehat{y}$ is a vector connecting the center of the meta-lens to each unit cell. In the above relationship, ${\theta }_{i}$, ${\varphi }_{i}$ are the spherical elevation and azimuth angle of incident waves, respectively.

**Figure 2:**
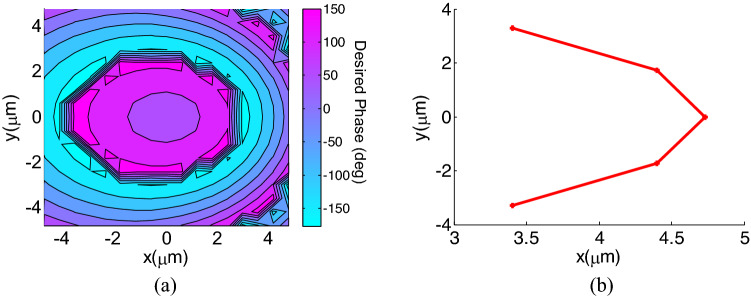
(**a**) Desired phase on the meta-lens when the reference nano-antenna is excited, (**b**) the optimum locations of the feeding nano-antennas at the $z=6.05 \, \upmu {\text{m}}$ plane.

On the other hand, the object wave ${\bar{\psi }}_{o}$, which is the output pattern in the desired direction, can be written as:

3 $${\bar{\psi }}_{o}\left({x}^{^{\prime}},{y}^{^{\prime}}\right)={A}_{o}\left(-\mathrm{cos}{\theta }_{o} \; cos{\varphi }_{o} \; \widehat{x}-\mathrm{cos}{\theta }_{o} \; sin{\varphi }_{o}  \; \widehat{y}\right) {e}^{j{k}_{0}(\overline{{R }^{^{\prime}}}. {\widehat{R}}_{o})}$$

where ${A}_{o}$ is the amplitude of the output wave and the far field vector ${\overline{R} }_{o}$ is written as ${\widehat{R}}_{o}=\mathrm{sin}{\theta }_{o}cos{\varphi }_{o} \widehat{x}+ \mathrm{sin}{\theta }_{o}sin{\varphi }_{o} \widehat{y}+ cos{\theta }_{o} \widehat{z}$, in which ${\theta }_{o}$, ${\varphi }_{o}$ determine the direction of the radiated beam. Finally, combining (1)–(3), the desired phase provided by the meta-lens is derived as:

4 $$\varphi \left({x}^{^{\prime}},{y}^{^{\prime}}\right)={k}_{o}\left(\mathrm{sin}{\theta }_{o} \; cos{\varphi }_{o} \; {x}^{^{\prime}}+\mathrm{sin}{\theta }_{o} \; sin{\varphi }_{o} \; {y}^{^{\prime}}\right)+{k}_{o}{\left({R}_{i}^{2}+{{x}^{^{\prime}}}^{2}+{{y}^{^{\prime}}}^{2}-2{x}^{^{\prime}}{R}_{i}\mathrm{sin}{\theta }_{i} \; cos{\varphi }_{i}-2{y}^{^{\prime}}{R}_{i}\mathrm{sin}{\theta }_{i} \; sin{\varphi }_{i}\right)}^{1/2}$$

where $\varphi \left({x}^{^{\prime}},{y}^{^{\prime}}\right)$ is the desired phase on the meta-lens that should be provided by the designed unit cells. As Eq. (4) illustrates, the phase provided by the metasurface is a function of ${R}_{i}$, the nano-antenna location, and also ${\theta }_{o}$, ${\varphi }_{o}$, the direction of the radiated beam. On the other hand, in our design, beam scanning is provided by switching the feeding nano-antennas. Therefore, for each radiation beam, ${\theta }_{o}$, ${\varphi }_{o}$ and ${R}_{i}$ vary resulting in different values for the phase profile on the metasurface, while our metasurface is not tunable and therefore its phase dose not dynamically change. To address this challenge, we define an error function representing the phase variation on the metasurface when the feeding antenna and radiation beam alters and minimize this function, by optimizing the location of feeding nano-antennas. For this purpose, the phase variation of the nano-antenna located at the center has been selected as the reference, and the locations of other feeding nano-antennas (${R}_{i},{\theta }_{i}$,${\varphi }_{i}$) are optimized to find the least phase difference with the reference antenna located at (${R}_{ir},{\theta }_{ir}$,${\varphi }_{ir}$). For simplicity, the parameters ${R}_{i},{\theta }_{i}$ are assumed to be fixed for all feeding nano-antennas (${R}_{i}=7.68\mu m,{\theta }_{i}={38}^{\circ}$ ) and we will only look for optimum values for ${\varphi }_{i}$. The error function which requires to be minimized is defined as:

5 $$Error\left({\varphi }_{i}\right)=\frac{1}{{N}_{x}{N}_{y}}\sum_{i=1}^{{N}_{x}{N}_{y}} \bigg[ \bigg(\frac{d\varphi \left({x}^{^{\prime}},{y}^{^{\prime}}\right)}{d{x}^{^{\prime}}} \big{|}_{{\varphi }_{i}}-\frac{d\varphi \left({x}^{^{\prime}},{y}^{^{\prime}}\right)}{d{x}^{^{\prime}}}\big{|}_{{\varphi }_{ir}}{)}^{2}+(\frac{d\varphi \left({x}^{^{\prime}},{y}^{^{\prime}}\right)}{d{y}^{^{\prime}}}\big{|}_{{\varphi }_{i}}-\frac{d\varphi \left({x}^{^{\prime}},{y}^{^{\prime}}\right)}{d{y}^{^{\prime}}}\big{|}_{{\varphi }_{ir}} \bigg )^{2}\bigg]$$

where ${N}_{x}, {N}_{y}$ represents the number of metasurface elements in the $x,y$ directions, respectively. The reference antenna is located on the x-axis (${\varphi }_{ir}={0}^{\circ}$) and other antennas incident angles (${\varphi }_{i}$) are calculated by minimizing the cost function and achieving minimum phase variation errors. For beam switching in 5 azimuth angles of (${\varphi }_{o}=[14{0}^{\circ},16{0}^{\circ},18{0}^{\circ},20{0}^{\circ},22{0}^{\circ}]$), the optimum locations are calculated and the results are shown in Fig. 2b, as ${\varphi }_{i}=[-43.{9}^{\circ},-21.{2}^{\circ},{0}^{\circ},21.{2}^{\circ},43.{9}^{\circ}]$. As illustrated in the Eqs. (1)–(4), the metasurface can be engineered to result in the desired far-field radiation pattern. Here, our goal for the radiation pattern has been to achieve a half power beam width of 20° and side lobe levels better than − 20 dBc. To achieve this goal, one of the key parameters is the size of the metasurface. In general, increasing the size of the radiating elements (metasurface here) results in narrower beams, however at the same time increases side lobe levels. Therefore, there is a tradeoff here. To achieve the aforementioned goals for the far-field pattern, we have optimized the metasurface dimensions and achieved the size of $10.2\times 10.2 \, \upmu {\text{m}}^{2}$ or $17\times 17$ unit cells for the designed metasurface. Using (4), the desired phase on this meta-lens, is calculated and the results are shown in Fig. 2a.

Achieving a uniform beam shaping with minimum radiation intensity fluctuations, is one of the most important challenges when designing Meta-lenses for beam steering applications. The reason behind this challenge is that since the phase and amplitude variations on metasurface are different for each of the feeding antennas^49–53^, choosing the optimum unit cell is a trade-off between desired reflection phases of each feeding antenna. To solve this problem, here we propose a novel optimization method based on weighted tapering unit cell selection. In this method, we define an error function based on the average square ratio of the radiation patterns of feeding antennas as:

6 $${Error}_{UC}\left({\varphi }_{i}\right)=\sum_{i=1}^{{N}_{feed}} \left( \frac{{Ef}_{i}^{2}\left({x}^{^{\prime}},{y}^{^{\prime}}\right)}{{Ef}_{r}^{2}\left({x}^{^{\prime}},{y}^{^{\prime}}\right)} \right ) (|{\varphi }_{desired, i}\left({x}^{^{\prime}},{y}^{^{\prime}}\right)-{\varphi }_{unitcells}|)$$

where ${N}_{feed}$ is the number of feeds, ${\varphi }_{desired, i}$ and ${\varphi }_{unitcells}$ represent the desired, and available (respectively) phase shifts on the metasurface, when the ${i}$th feeding antenna is used. Also, $E{f}_{i}$, $E{f}_{r}$ indicate the electric field amplitudes (on the metasurface) for the ${i}$th feeding antenna and the reference antenna, respectively. Using (6), the desired phase for each unit cell, from any feeding antenna, is optimized to achieve the total minimum weighted error ${Error}_{UC}$ at the wavelength of 1550 nm. Therefore, the selection of Meta-lens unit cells in the proposed method, is a compromise between the desired radiation patterns.

**Figure 3:**
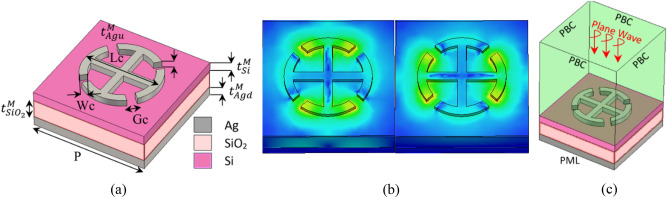
(**a**) Perspective view of the proposed unit cell. (**b**) Electric-Field response of the unit cell for TM (left) and TE (right) modes at the wavelength of 1550 nm. (**c**) Simulation setup of the structure. Plane wave excitation and periodic boundary conditions, are utilized to calculate the reflection phases of each unit cell element.

Now that the required phases are determined, we need to select an appropriate unit cell to provide the suitable phase shift. The proposed unit cell is shown in Fig. 3a. As shown in this figure, it consists of four layers in which silicon and silicon dioxide layers are sandwiched between two silver layers. The silver layer at the bottom reflects the light and avoid it to go through the structure. The Upper silver layer formed by two similar arms and three variable parameters, provide different reflective phases. This symmetry of the unit cell caused it to show similar behavior for different polarizations of the incoming light. This behavior is shown in Fig. 3b, where the response of the cell is shown for both TM and TE polarizations of the incoming light at the wavelength of 1550 nm. Due to the fact that a large part of the return waves is concentrated inside the silicon and silicon dioxide layers, the loss of the unit cell has been reduced dramatically. Another important point is the repetitive period of the unit cell. According to the theory of periodic surfaces and Floquet waves^54,55^, in order to avoid the grating lobes, the dimensions of the cell, should be smaller than:

7 $$p<\frac{{\lambda }_{0}}{(1+|sin{\theta }_{i max}|){ n}_{d}}$$

In the above relation, $p$ is the repetitive period of the unit cell, ${\theta }_{imax}$ is the maximum incident angle respect to the unit cell, and ${n}_{d}$ is the refractive index of the surrounding material which is silicon dioxide here, with (${n}_{d}=1.45$). Considering that ${\uptheta }_{imax}\cong {50}^{\circ}$, period has been selected as $p=600 nm$ ($0.39\lambda$ at the wavelength of 1550 nm). The other parameters of the unit cell are illustrated in Table 1. As shown in this table, the proposed unit cell has three variable parameters, *Wc, Lc*, and *Gc*, making 204 types of cells to achieve different phase variation on the meta-lens. Due to the complexity of the proposed structure, full-wave CST software^60^ has been used to simulate reflected waves from unit cells. In this simulation, the unit cell is placed in a SiO_2_ environment and the periodic boundary conditions (PBC) are applied around the cell. Figure 3c shows the simulation setup used for the cell characterization.

**Table 1:** Geometrical parameters of the proposed unit cell.

Value (nm)	Parameter	Value (nm)	Parameter
20	${t}_{Agu}^{M}$	600	$P$
10	${t}_{Si}^{M}$	[30 50 70]	Wc
100	${t}_{{SiO}_{2}}^{M}$	220:10:580	Lc
50	${t}_{Agd}^{M}$	[50 100]	Gc

**Figure 4:**
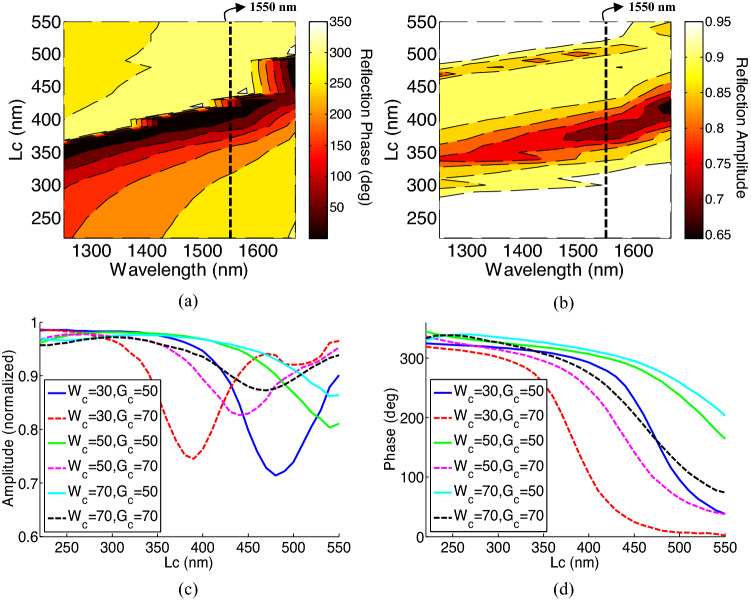
(**a**) Phase and (**b**) Amplitude ratio of the reflected wave as a function of wavelength and *Lc*. Reflection (**c**) amplitude, and (**d**) phase versus geometrical parameters at the wavelength of 1550 nm. The unit used for all geometrical parameters is nm.

Figure 4a and b, show the reflected amplitude and phase of the unit cell versus wavelength and for different values of *Lc*. As shown in this figure, by alternating *Lc*, a range of ${0}^{\circ}$ to ${320}^{\circ}$ phase shift can be covered at the wavelength of 1550 nm. Furthermore, according to the results of this figure, the reflection amplitude is higher than 0.7. Figure 4c and d compare the reflection phase and amplitude versus other parameters at the wavelength of 1550 nm. As shown in this figure, tuning the *Gc* and *Wc* provides different phase gradients and can be used to realize any phase variations on the mat-lens unit cells. Also, the fact that all unit cells can support the reflection amplitude of 0.7 or higher, makes it possible to achieve a high efficiency for the designed meta-lens.

**Figure 5:**
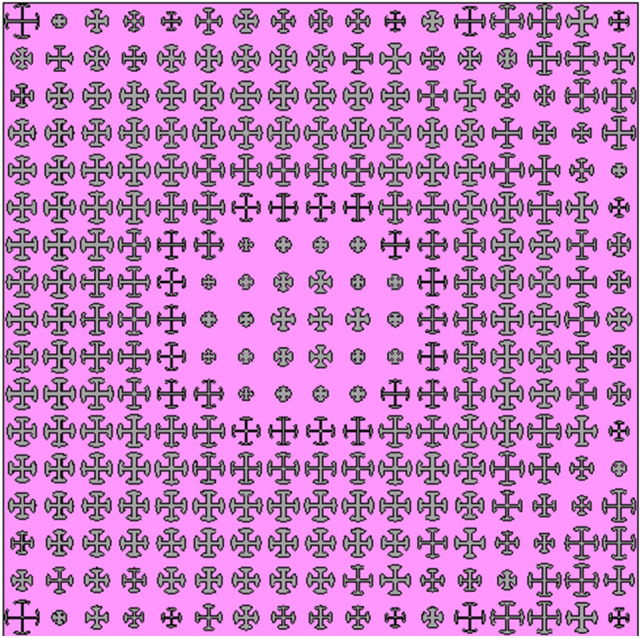
Front view of the designed meta-lens.

**Figure 6:**
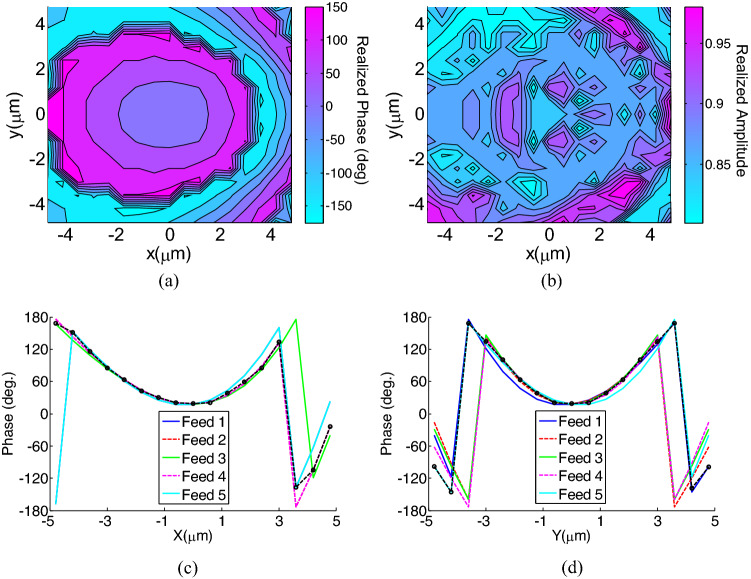
Realized reflection (**a**) phase, (**b**) amplitude, (**b**) of the designed meta-lens. The realized phase (shown with dots) is compared with the desired phase required for each feeding antenna (**c**) at the x = 0 and (**d**) at the y = 0 planes.

Finally, using Eq. (6), and results shown in Fig. 4, the metasurface unit cells are selected to achieve a uniform beam scanning. The proposed meta-lens is shown in Fig. 5. The realized reflection amplitude and phase of the designed meta-lens are shown in Fig. 6a and b, respectively. According to this figure, the designed metasurface reflects more than 80% of the incident waves, providing a high efficiency for the whole antenna system. In Fig. 6c and d, the realized phase on the meta-lens is compared with the desired phases on it for each radiation angle. According to these figures, the phase errors are increased at the edges of the metasurface, however this only can affect the side lobe levels in the radiation pattern.

### Feeding nano-antennas

**Figure 7:**
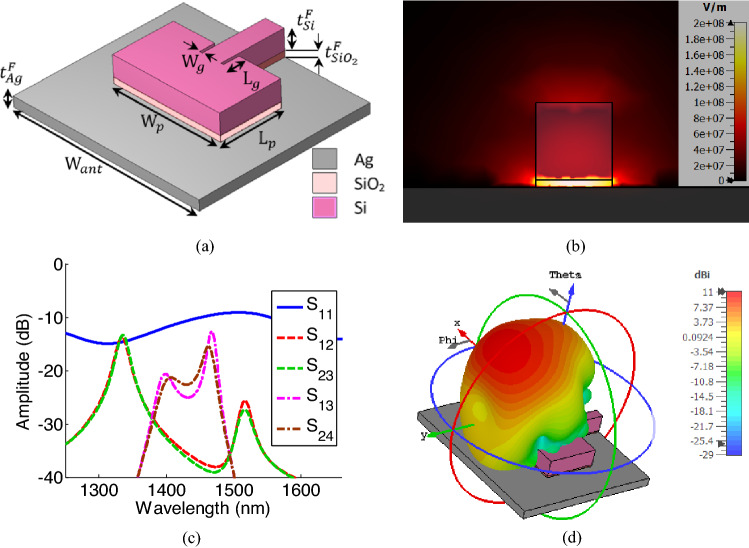
(**a**) Perspective view of the designed feeding nano-antenna with dimensions of ${{t}^{F}}_{Ag}=100 {\text{nm}}, \, {{t}^{F}}_{{SiO}_{2}}=20 {\text{nm}}, \, {{t}^{F}}_{Si}=150 {\text{nm}}$, $W\_ant=2000 \, {\text{nm}},$ ${W}_{p}=1150 \, {\text{nm}}, {L}_{p}=640 \, {\text{nm}}, { W}_{g}=10 \, {\text{nm}}, {L}_{g}=160 \, {\text{nm}}$ (**b**) Dominant TM mode of the feeding nano-antenna in which light is highly confined inside the thin SiO_2_ layer. (**c**) Scattering parameters of the feeding nano-antennas. The return loss of Antenna#1 (S_11_) along with the coupling between nano-antennas are shown in this figure. (**d**) Far 3D radiation pattern of the feeding nano-antenna at the wavelength of 1550 nm.

A perspective view of the feeding nano-antenna is shown in Fig. 7a. The proposed nano-antenna is designed based on hybrid plasmonic structures, in which a low refractive index layer (silicon dioxide, here) is sandwiched between a metal (silver, here) and a high refractive index dielectric (silicon, here)^5–8^. Figure 7b illustrates the mode excited inside the waveguide feeding the designed nano-antenna. As shown in this figure, the light is confined inside the thin SiO_2_ layer as expected for hybrid plasmonic structures^5–8^. The feeding nano-antennas are designed based on the model developed in^5^ for antennas fed by plasmonic waveguides. As proved in^5^, in the hybrid plasmonic waveguide feeding the nano-antenna, the tangential component of the Electric field along the propagation direction is much smaller than its normal component, and therefore the TM mode excited in the waveguide can be approximated with a TEM mode. This makes it possible to use the transmission line theory to precisely model and design the structure^5^. For the best matching, the width of the hybrid plasmonic line is considered to be the same as graphene switch and equal to ${w}_{l}=100 \, {\text{nm}}$, and to have a high confinement inside the thin SiO_2_ layer, the thickness of this layer, ${t}_{Si{o}_{2}}^{F}$ is chosen to be equal to 20 nm. For the length, ${L}_{p}$, and the width, ${w}_{p}$, of the plasmonic hybrid patch, we have used the transmission line model developed in^5^, to get initial values for the design and then slightly tuned these parameters to achieve the desired pattern for the antenna. Finally, the size of the inset part, ${L}_{g}$ and ${W}_{g}$ are selected based on the technique explained in^25^. The inset part is used to provide better impedance matching between the nano-antenna and the feeding hybrid plasmonic waveguide.

The designed nano-antennas are then placed at optimum locations calculated by the Eq. (5). The scattering parameters (S-Parameters) of feeding nano-antennas versus the wavelength are shown in Fig. 7c. In this figure, the return loss of the feeding nano-antenna#1 (see Fig. 1a) along with the mutual coupling between nano-antennas are shown. As shown in this figure, the illustrated return loss is less than − 9 dB showing a good impedance matching for the designed antenna. Furthermore, the results of this figure show that there is a low mutual coupling between the designed antennas (less than − 12 dB). In Fig. 7d, the radiation pattern of the designed nano-antenna is shown. According to this result, the proposed feeding nan-antenna has a high directivity of 10.2 dB and an angle deviation of ${38}^{\circ}$ (respect to the z-axis). This is the reason behind arranging the feeding antennas at an angle of ${38}^{\circ}$ respect to the meta-lens.

### Graphene-based switchable power divider

**Figure 8:**
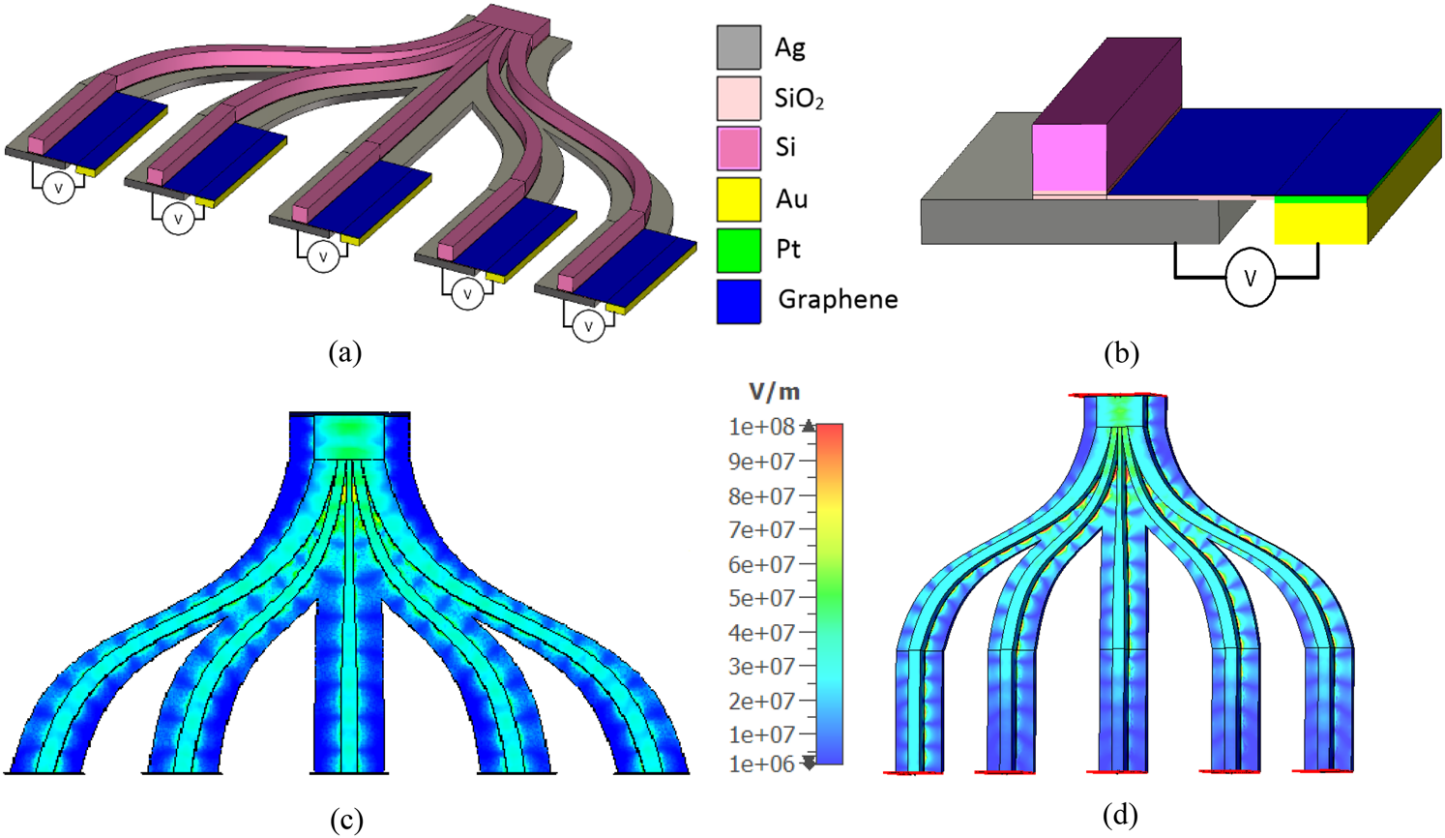
(**a**) Perspective view of the designed switchable power divider, (**b**) Perspective view of the biasing circuit used to apply desirable voltage to Graphene sheets, (**c**) Electric field intensity on the power divider at the wavelength of 1550 nm, when (**c**) all branches are switched ON (**d**) only one branch is switched ON and others are switched OFF.

In the proposed method, the rotation of the beam is obtained by choosing among feeding nano-antennas. Therefore, a controllable power divider is required to select among antennas. Figure 8a shows the structure of the designed graphene-based switch. As shown in this figure, the switch is also designed based on hybrid plasmonic structure, in which a layer of silicon dioxide with a thickness of 20 nm is sandwiched between two layers of silver and silicon with thicknesses of 100 nm and 150 nm, respectively. Also, the line width of 800 nm has been selected for the feeding waveguide.

Due to the non-uniform distribution of the field across the width of the feeding waveguide, asymmetric line widths have been used to divide the fields into the branches, equally. The distance between the dividing branches is also chosen according to the location of the feeding nano-antennas. The graphene sheet is located in the middle of the SiO_2_ layer, and is connected to the biasing voltage through the circuit shown in Fig. 8b. Voltage stimuli enables the variation of chemical potential of graphene sheets resulting in modulation of conductivity of graphene^25^. The maximum loss at the wavelength of 1550 nm, (OFF state of the switch) can be obtained when the chemical potential of graphene is equal to 0.51 eV, and the minimum loss (ON state of the switch) can be obtained for a chemical potential of 0 eV on the graphene sheet^25^.

The numerically calculated electric fields on the proposed power divider are shown in Fig. 8c and d. Figure 8c shows the results when all output branches are switched ON, while Fig. 8d shows the results when only one branch is switched ON and others are OFF.

## Results and discussion

**Figure 9:**
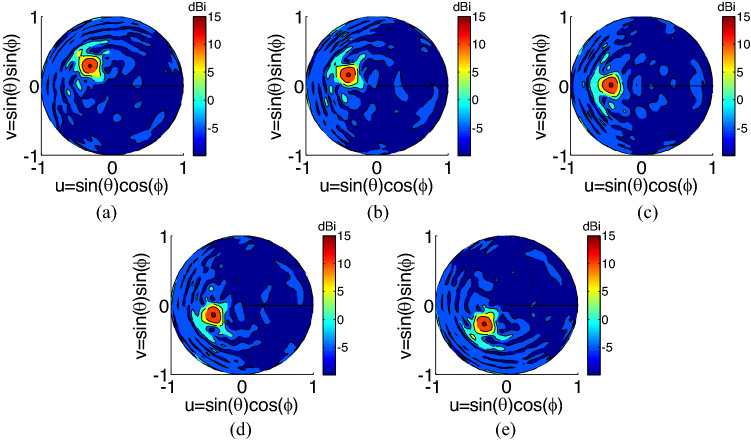
The radiation pattern of the device in the u–v plane, when different feeding nano-antennas are selected. Beam steering is clearly shown in this figure.

**Figure 10:**
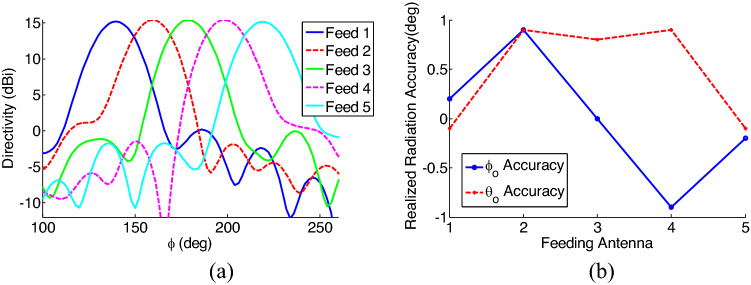
(**a**) 2D radiation pattern of the device at ${\theta }_{o}={38}^{\circ}$, for different feeding antennas. (**b**) Realized radiation accuracy achieved when different feeding nano-antennas are chosen. Both figures show the results at the operation wavelength of 1550 nm.

In this section, we investigate the performance of the proposed device by numerically analyzing the whole structure. The numerical simulation is performed using CST full software^60^. In this simulation, the far field radiation pattern of the reflecting metasurface is calculated by solving integral equations using Method of Moment, and for the excitation the nano-antennas radiation pattern (calculated using Finite Element Method and shown in Fig. 7d) is used. The results of this simulation are shown in Figs. 9 and 10. Figure 9 shows the radiation pattern in the u–v plane, when different nano-antennas are excited. A u–v plane is a geometric plane to show the 3D pattern in the 2D circular figure. In the u–v plane, the axes are defined as $u=\mathrm{sin\theta }cos\varphi , v= \mathrm{sin\theta sin\varphi }$. As shown in this figure, the beam steering is realized by switching between feeding antennas. Furthermore, the results of this figure shows that a Directivity of 15 dBi is achieved for the device. For more clarification on the resultant pattern, Fig. 10 a, shows the radiation pattern in 2D format (for a fixed $\theta$, and different values of $\varphi$) which illustrates the beam steering more clearly. Furthermore, the results of this figure shows that there is a low variation (less than 1 dB) in the achieved Directivity for all different beam directions which is an important advantage of this work when compared to previously reported works on optical beam steering^49–55^. This figure also shows that a side lobe level of less than 15 dB is achieved for all different beam angles which is another advantage of the proposed structure.

**Figure 11:**
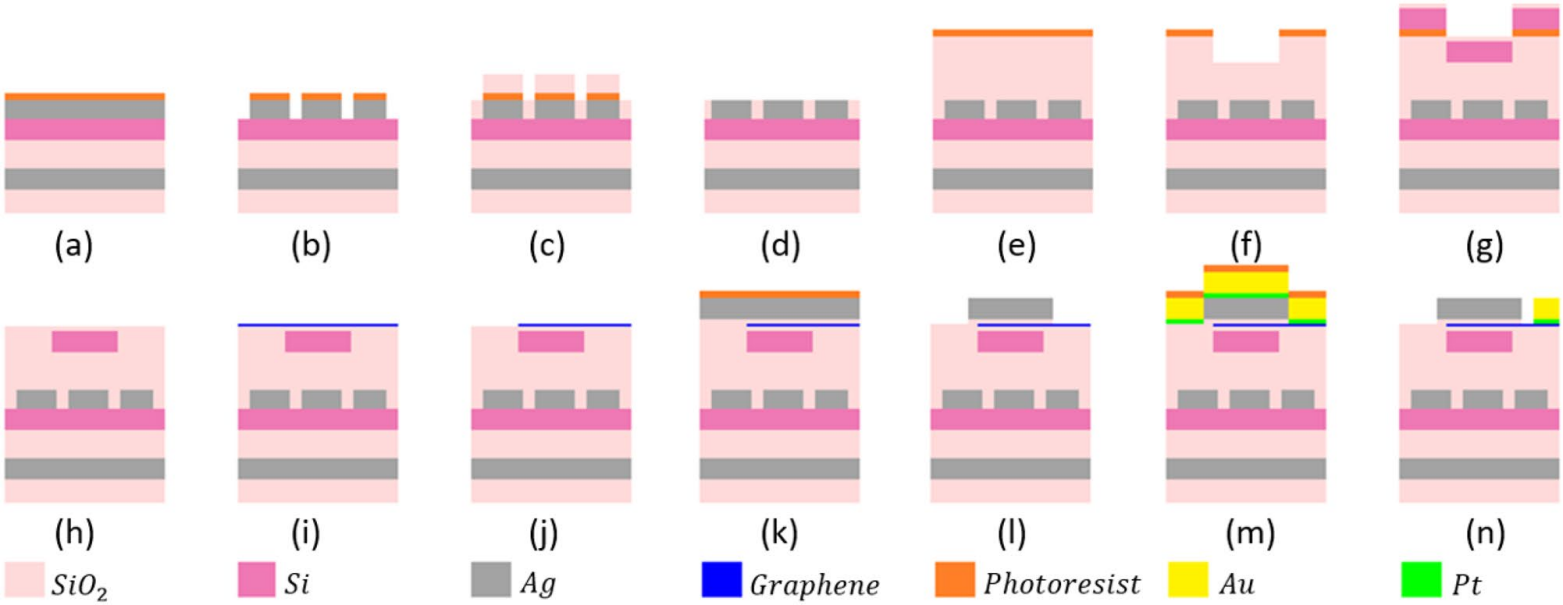
The suggested fabrication procedure for the proposed structure. (**a**–**d**) illustrating the fabrication process of the proposed metasurface: (**a**) Metasurface layers (Ag, SiO_2_, Si, and again Ag) are deposited on the substrate (**b**) The top Ag layer is patterned to provide unit cells of the metasurface using electron beam lithography (EBL) and plasma etching (**c**) The SiO_2_ layer is deposited to fill between the unit-cells. (**d**) Resolving the resist in a solvent and removing the excess materials. (**e**–**n**) illustrating the fabrication process of the proposed nano-antennas, switch and divider: (**e**) The SiO_2_ layer is deposited on the fabricated metasurface. (**f**) EBL and plasma etching procedure are applied to prepare the structure for the desired shape of nano-antenna, divider and switch. (**g**) The 150 nm Silicon layer and a 10 nm SiO_2_ layer are deposited. (**h**) Resolving the resist in a solvent and removing the excess Si and SiO_2_. (**i**) Graphene with the catalyst layer is transferred on the SiO_2_ layer. (**j**) The laser beam creates patterns in the catalyst layer and allows for patterning the graphene layer. (**k**) The 10 nm SiO_2_ and 100 nm Ag layers are deposited. (**l**) EBL and plasma etching procedures are applied to pattern the Ag and SiO_2_ materials in order to create nano-antennas, divider and switch. (**m**) The platinum and gold layers are deposited. (**n**) EBL and plasma etching procedure are applied to pattern the Au and Pt layers and to create the biasing circuit of the switchable power divider.

Accuracy of proposed method to achieve the desired radiation angles (${\theta }_{o}={38}^{\circ}, \, {\varphi }_{o}=[14{0}^{\circ},\, 16{0}^{\circ},\,18{0}^{\circ}, \, 20{0}^{\circ}, \, 22{0}^{\circ}]$) is shown in Fig. 10b. This figure shows the difference between the desired direction and achieved direction. As shown in this figure, the difference for both ${\theta }_{o}, {\varphi }_{o}$ is less than ${1}^{\circ}$ illustrating a very good accuracy for the proposed device. A suggested fabrication procedure for the proposed device is shown in Fig. 11. As shown in this figure, the fabrication procedure has 3 steps to fabricate metasurface, nano-antennas and switchable power divider and finally the biasing circuit. As illustrated in this figure, the proposed device can be fabricated using standard nano-fabrication techniques.

**Figure 12:**
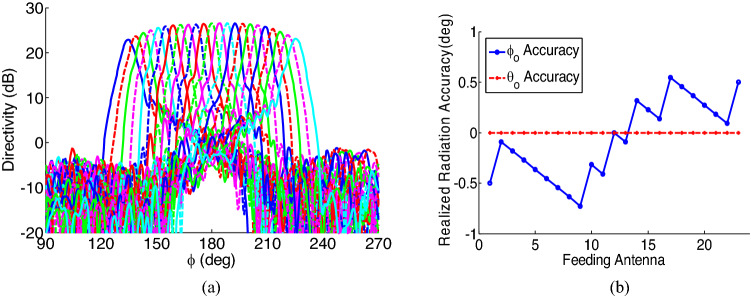
(**a**) 2D radiation pattern of the extended version of the device with 23 feeding nano-antennas (**b**) Realized radiation accuracy achieved for the extended version of the device when different feeding nano-antennas are chosen.

In order to illustrate the ability of the proposed method to be extended in order to achieve a narrower beam, and higher resolution in the beam steering, here we report the results for a device with 23 feeding nano-antennas, whose locations are optimized using (5). For this design, the metasurface dimensions are considered to be $39\times 39 \mu {m}^{2}$, and the optimum unit cells are selected to achieve minimum phase error calculated using (6). The resultant radiation patterns, calculated using the Fourier transform method^56^, are shown in Fig. 12a. As shown in this figure, the beam steering is achieved for angles in the range of $-{45}^{\circ}:{45}^{\circ}$, with 23 steps. Furthermore, these results show that a half-power beam width of ${5}^{\circ}$ is achived in the extended version of the device. Figure 12b, shows the accuracy of the radiation pattern illustrating a high accuracy for the proposed method.

## Conclusion

A new integrated optical beam steering device was proposed and investigated. The proposed device consists of a meta-lens illuminated by five switchable feeding nano-antennas, all integrated inside a SiO_2_ medium. To achieve a high accuracy in the direction of radiated beams, low side-lobe levels (SLL), and low fluctuations in the radiation intensity, analytical algorithms were developed and utilized to optimize the location of feeding nano-antennas and also unit cells constructing the meta-lens. Numerical full-wave simulation results showed that the device has a directivity of better than 15 dBi for all five radiation angles in field of view of 100 degrees, a radiation angle accuracy of better than one degree and SLL of better than 15 dB. An extended version of the proposed device with 23 feeding nano-antennas was also designed and analyzed. The results of the extended version of the device also showed a high accuracy and low fluctuations in the radiation intensity illustrating the ability of the design methodology to be used for extended devices. The proposed device can be used in many optical applications from multi focusing optical communication systems to highly integrated LIDAR systems.

## Methods

In order to analyze the performance of the whole structure, a 3D full-wave numerical simulation was performed using CST software^60^ and the Uni-directional simulation setup was used for it. In this simulation, the proposed structure was placed into the SiO_2_ background and the boundary conditions were defined as open-add-space (modeling the radiation condition). The simulation was performed in two steps. In the first step, the nano-antennas, and graphene-based switchable power divider were excited using a waveguide port and the structure was analyzed using Finite Elements method. In the step two, the meta-lens was illumined with the electric field radiation pattern achieved from results of step one. In this part, the total radiation pattern of the device was extracted using the method of moments.

## Data Availability

The dataset used and/or analyzed during the current study are available from the corresponding author on reasonable request.
